# Early Diagnosis of Multiple Sclerosis Using Swept-Source Optical Coherence Tomography and Convolutional Neural Networks Trained with Data Augmentation

**DOI:** 10.3390/s22010167

**Published:** 2021-12-27

**Authors:** Almudena López-Dorado, Miguel Ortiz, María Satue, María J. Rodrigo, Rafael Barea, Eva M. Sánchez-Morla, Carlo Cavaliere, José M. Rodríguez-Ascariz, Elvira Orduna-Hospital, Luciano Boquete, Elena Garcia-Martin

**Affiliations:** 1 Biomedical Engineering Group, Department of Electronics, University of Alcalá, 28801 Alcalá de Henares, Spain; almudena.lopez@uah.es (A.L.-D.); rafael.barea@uah.es (R.B.); carlo.cavaliere@uah.es (C.C.); jmr.ascariz@uah.es (J.M.R.-A.); 2 Computer Vision, Imaging and Machine Intelligence Research Group, Interdisciplinary Center for Security, Reliability and Trust (SnT), University of Luxembourg, 4365 Luxembourg, Luxembourg; miguel.ortizdelcastillo@uni.lu; 3 Miguel Servet Ophthalmology Innovation and Research Group (GIMSO), Department of Ophthalmology, Aragon Institute for Health Research (IIS Aragon), Miguel Servet University Hospital, University of Zaragoza, 50018 Zaragoza, Spain; mariasatue@gmail.com (M.S.); mariajesusrodrigo@hotmail.es (M.J.R.); elvira.orduna@gmail.com (E.O.-H.); 4 Department of Psychiatry, Hospital 12 de Octubre Research Institute (i+12), 28041 Madrid, Spain; evamas01@ucm.es; 5 Faculty of Medicine, Complutense University of Madrid, 28040 Madrid, Spain; 6 Biomedical Research Networking Centre in Mental Health (CIBERSAM), 28029 Madrid, Spain

**Keywords:** multiple sclerosis, optical coherence tomography, convolutional neural network, generative adversarial network

## Abstract

Background: The aim of this paper is to implement a system to facilitate the diagnosis of multiple sclerosis (MS) in its initial stages. It does so using a convolutional neural network (CNN) to classify images captured with swept-source optical coherence tomography (SS-OCT). Methods: SS-OCT images from 48 control subjects and 48 recently diagnosed MS patients have been used. These images show the thicknesses (45 × 60 points) of the following structures: complete retina, retinal nerve fiber layer, two ganglion cell layers (GCL+, GCL++) and choroid. The Cohen distance is used to identify the structures and the regions within them with greatest discriminant capacity. The original database of OCT images is augmented by a deep convolutional generative adversarial network to expand the CNN’s training set. Results: The retinal structures with greatest discriminant capacity are the GCL++ (44.99% of image points), complete retina (26.71%) and GCL+ (22.93%). Thresholding these images and using them as inputs to a CNN comprising two convolution modules and one classification module obtains sensitivity = specificity = 1.0. Conclusions: Feature pre-selection and the use of a convolutional neural network may be a promising, nonharmful, low-cost, easy-to-perform and effective means of assisting the early diagnosis of MS based on SS-OCT thickness data.

## 1. Introduction

In recent years, the usefulness of deep learning (DL) techniques has been demonstrated in many applications, including in medicine [1]. Most medical applications have been in specialties related to diagnostic imaging, such as radiology or dermatology [1,2,3,4,5,6], in genomics [7,8] and, in lower numbers, in one-dimensional signal analysis, such as electroencephalograms (EEG) [9,10,11,12,13], electrodermal activity [14] and electrocardiograms (ECG) used in arrhythmia classification [15,16].

Several recent reviews analyze ophthalmologic applications of DL [17,18]. These include the diagnosis and classification of glaucoma based on disc photos, the segmentation of retinal layers using optical coherence tomography (OCT), forecasting future Humphrey visual fields [19], the diagnosis of diabetic retinopathy [20], macular degeneration progression [21], retinopathy of prematurity [22] or estimation of retinal sensitivity in macular telangiectasia type 2 [23].

Convolutional neural networks (CNNs) are one of the solutions widely used in DL. CNNs are inspired by the structure of the human visual system [24]. Like the visual system, CNNs are arranged in layers of increasing complexity. The way in which CNNs analyze a group of pixels in an image is similar to how the receptive fields of the retina operate. Both the group of pixels and the receptive fields detect image features, such as the direction, edges and movement, within a region. The cortical cell complexity increases with image complexity, just as it does in CNNs, in which information is analyzed up to the upper layer of the artificial neural network (which would be the brain).

Broadly speaking, CNNs perform two functions: they extract the most descriptive features (feature maps) in the input images using convolutions (feature module: FM) and implement the classification with an artificial neural network (classification module: CM). The FM is made up of one or more layers, which, by means of convolutions with predetermined filters or kernels, aim to obtain the spatial correlative features (curves, edges, intensity gradients, etc.) that identify the information in the input images with maximum discriminant capacity. In the FM, it is common to perform other types of processing (nonlinear transformations, polling) that can improve the learning convergence speed and reduce the possibility of overfitting. The number of FMs used in a particular problem depends on its complexity. The classification module is usually a feed-forward neural network that has the FM output as its inputs and in which, through supervised training (e.g., variants of the backpropagation algorithm), the weights are adjusted [25].

The retina is an extension of the central nervous system (CNS), and alterations to its functioning or structure can precede manifestations of several disorders, among them multiple sclerosis (MS), Parkinson’s disease, bipolar disorder, etc. Structural analysis of the retina using optical coherence tomography (OCT) has demonstrated its utility as an accessible biomarker in MS, diabetic retinopathy, glaucoma, Parkinson’s, etc. [26,27,28,29,30,31,32]. The OCT technique is based on low-coherence interferometry using a near-infrared optical laser that exploits the light-scattering phenomenon. Swept-source OCT (SS-OCT) uses a short-cavity swept laser with a tunable wavelength centered at ~1 µm (sweeping range of approximately 100 nm) [33]. The typical scanning rate is 100,000 A-scans/s, thereby achieving an acceptable reduction in motion artifacts. Another important feature of SS-OCT is that, because it typically operates at wavelengths between 1060 nm and 1310 nm, it achieves greater penetration in retinal tissue than preceding OCT technologies that operate at approximately 850 nm. Using OCT, it is possible to obtain, in vivo, noninvasively and with great precision, near-histological images of the retina and measurements of the thicknesses of the various layers.

DL methodologies are often used to perform anatomical segmentation of the various layers of the retina [34,35,36]. Following specific protocols, quantitative information on neuroretinal thickness is transferred to specialist practitioners to support their clinical decisions (e.g., mean value of the thickness of a retinal layer in a region defined by the ETDRS (early-treatment diabetic retinopathy study scan) chart [37], mean value of the thickness in a 3.5 mm-diameter circular scan around the head of the optic nerve, etc.). Traditional methods of analyzing retinal layer thicknesses obtained using OCT are based on extracting the features from those images and then classifying them using, for instance, a support vector machine (SVM) [30]. In contrast, DL directly processes the images in the convolutional layers and implements a predictive model based on the features extracted. It eliminates the need to hypothesize a detection model, instead providing an end-to-end solution spanning the input OCT images through to the diagnostic decision.

MS is a chronic neurodegenerative demyelinating disease that affects the CNS and causes a wide variety of symptoms (cognitive, motor, visual, etc.) in patients. In 2015, it affected 2.5 million people worldwide. In a high percentage of sufferers, the onset of the disease occurs between the ages of 20 and 30. It affects approximately two females for every male. MS is diagnosed using the McDonald criteria [38], which are principally based on the integration of clinical, imaging and laboratory findings. However, the McDonald criteria are only applicable when the disease is at a fairly advanced stage.

In medicine, a biomarker is a characteristic that can be objectively measured and evaluated as an indicator of normal biological processes, pathogenic processes or pharmacological responses to therapeutic intervention [39]. We are working on the development of new tools that help neurologists to expedite MS diagnosis as it is traditionally a lengthy process involving several invasive tests such as magnetic resonance imaging, or lumbar puncture to obtain cerebrospinal fluid for analysis. The use of a noninvasive test that serves as a diagnostic biomarker would reduce the need for many invasive tests and would lead to the earlier diagnosis of clinical certainty and, therefore, to earlier initiation of treatment for patients, thus improving their quality of life and minimizing the impact of the disease on their health.

In addition, misdiagnosis of MS is relatively frequent, mainly because many clinical syndromes mimic MS. Syndromes incorrectly identified as typical of MS include complete transverse myelopathy, intractable vomiting/nausea/hiccoughs and bilateral optic neuritis/unilateral optic neuritis with poor visual recovery [40]. For example, [41] describes how 18% of new MS patients at two clinics were discovered to be misdiagnosed, while [42] identifies misdiagnosis in 8 out of 112 cases (7.1%). The diagnostic delay from symptom onset to MS diagnosis can range from 7 [43] to 30 months [44], which can have a negative impact on disease trajectory.

Research into MS biomarkers that enable early diagnosis and objective evaluation of the treatment is a priority if patients are to benefit from appropriate and effective therapy as soon as possible. The emergence of effective disease-modifying treatments has created an impetus to diagnose as early as possible because the use of such treatments has contributed to improved longevity and to reduced rates of both worsening and development of secondary progressive multiple sclerosis [45].

The machine-learning techniques applied to MS have mainly targeted early diagnosis, including the analysis of potential conversion of possible preliminary stages of the disease, such as radiologically isolated syndrome, into definitive MS [46], or the prediction of disease progression and outcomes [47]. The type of data analyzed and the analysis and classification tools employed vary between studies. By way of example, in [46], multifocal visual-evoked potential features are classified with an RUSBoost boosting-based sampling algorithm; [47] analyzes clinical information (age, onset age, initial MS manifestations, and clinical and examination findings that led to the diagnosis, such as MRI, evoked potentials, etc.) using four classifiers (support vector machine (SVM), k-nearest neighbors (k-NN), a decision tree, and linear regression); or [48], which uses recurrent neural networks to predict disability progression in MS patients over a two-year horizon. In a recent paper, [49] investigates use of machine learning techniques to diagnose and predict the course of disability in MS. Only retinal nerve fiber layer (RNFL) thickness data are analyzed (mean thickness in each of the four quadrants into which the peripapillary area is divided) and data such as age, sex, best-corrected visual acuity, etc. are taken into account. These are not newly diagnosed patients, as the MS duration (years) = 10.15 ± 8.30. For MS diagnosis, the authors used nine features, and the best result was obtained with an ensemble classifier (accuracy = 87.7%), while for MS disability course prediction, the best predictor was a long short-term memory recurrent neural network (accuracy = 81.7%).

In 1999, it was verified using the OCT technique that there is a significant reduction in the retinal nerve fiber layer (RNFL) in patients with MS when compared with control subjects [50]. Subsequent studies have explored the advantages of OCT as a valid biomarker in the assessment of MS [26,51,52], and its diagnostic capability in MS is the subject of ongoing research. Patients with early-stage or moderate MS have about 10 microns less average RNFL thickness than healthy subjects of the same age and sex [53].

In many clinical studies, it is not possible to obtain the massive and diverse volumes of data that deep-learning training requires to ensure there is no overfitting. To alleviate this data scarcity, one possible solution is to use generative adversarial networks (GANs). Since the introduction of the GAN approach by Goodfellow et al. [54], the technique has demonstrated its ability to learn data distributions and generate artificial data. In recent years, GANs have been extensively applied to a great variety of real-world applications [55,56,57,58]. In medicine, GANs have been used in image synthesis [59] and radiology applications [60], or to generate one-dimensional medical signals, such as EEGs [61,62] or ECGs [63]. Concerning GANs’ applications in diagnosis of MS, papers that can be cited include [64], in which the number of images is increased to analyze brain structural connectivity, or [65], in which the authors verify that performing data augmentation on a variety of T1-weighted MRIs improves both tissue and lesion segmentation in MS.

However, to our knowledge, there are no papers evaluating the application of a DL system to MS early diagnosis based on OCT measurements. OCT equipment can obtain a high-density grid of thickness measurements for most layers of the retina. This 2D information can then be used as an input to a DL system.

Taking into account that the retina is part of the central nervous system, our working hypothesis is that the demyelination and inflammation processes characteristic of MS can be detected in the disease’s early stages by analyzing the thicknesses obtained by OCT and assessing them using computational intelligence.

In this context, the objective of this study is to diagnose early MS using CNNs fed with images of retinal layer thicknesses obtained with SS-OCT. In order to augment the set of CNN training images, synthetic images of retinal thicknesses are generated using GANs.

The main contributions of this paper can be summarized as follows: first, we propose applying CNNs to the diagnosis of early-stage MS using the most discriminant retina layer thicknesses measured by OCT; to our knowledge, this is the first time that a CNN has been used to classify retinal layer thickness data obtained using OCT. Second, in order to possess a comprehensive CNN training database, data augmentation was performed using a deep convolutional generative adversarial network. Third, we developed a powerful tool for diagnosing MS based on a robust validation method, although this needs to be confirmed in further studies with different populations.

## 2. Materials and Methods

### 2.1. Patient Database

The principles of the Declaration of Helsinki were applied in this study. The study protocol was approved by the Clinical Research Ethics Committee of Aragón (CEICA, Zaragoza, Spain). Written informed consent was obtained from all individuals.

Based on the McDonald criteria [66], a neurologist specialized in MS diagnosed relapsing-remitting MS (RRMS) in the patients. Taking into account the Expanded Disability Status Scale (EDSS) [67] and the treatment received by each patient, the degree of disability was quantified. The EDSS scale ranges from 0 to 10 in 0.5-unit increments that represent progressively higher levels of disability (0 = normal neurological examination with no MS-related limitation, 10 = death due to MS).

All subjects underwent complete neuro-ophthalmic examination: (a) evaluation of Snellen best-corrected visual acuity; (b) evaluation of contrast sensitivity vision (CSV) with the CSV1000 test at 3, 6, 9 and 12 cycles per degree; (c) assessment of color vision using the 38 Ishihara plates (Gima Professional Medical Products, Gessate, Italy, 2002); (d) ocular motility test using cover–uncover tests; (e) pupillary light/dilation reflex test by the neuro-ophthalmologist; (f) anterior segment examination (with slit-lamp examination); (g) measurement of intraocular pressure (IOP) using Goldmann applanation tonometry; and (h) fundoscopic examination using a Topcon ID10 indirect ophthalmoscope (Topcon corporation, Tokyo, Japan).

Subjects were excluded from the study if they met any of the following conditions: (a) concomitant ocular disease, previous history of retinal pathology, glaucoma, amblyopia or systemic conditions that could affect the visual system; (b) visual acuity < 0.6 (Snellen chart scale 20/200), eyes longer than 25.2 mm and refractive errors ≥5 diopters (D) of equivalent spherical diameter or ≥3D of astigmatism, intraocular pressure > 20 mmHg, or history of optic neuritis; (c) active MS flare (or any neurological deficit in the 6 months prior to enrollment) to avoid masking of neural damage by acute axonal loss.

### 2.2. OCT Method

Ocular structures were measured using the Topcon Deep Range Imaging Triton (DRI-OCT. Topcon, Tokyo, Japan) device, which implements the 3D Wide protocol. This equipment performs multimodal scanning source OCT with a nonmydriatic color fundus camera (invisible light source wavelength of 1050 nm, spectral bandwidth of 100 nm, scanning speed of 100,000 A-scans per second, axial resolution of 8 μm). The 3D Wide protocol scans an area of 12 × 9 mm^2^, including both the macular and peripapillary areas, and obtains a total of 45 × 60 measurement points for each of the structures.

**Figure 1:**
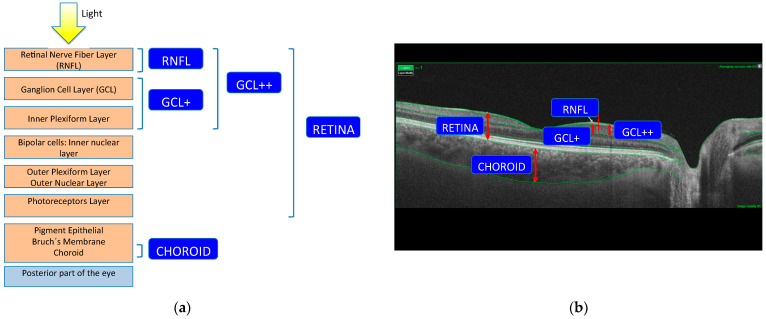

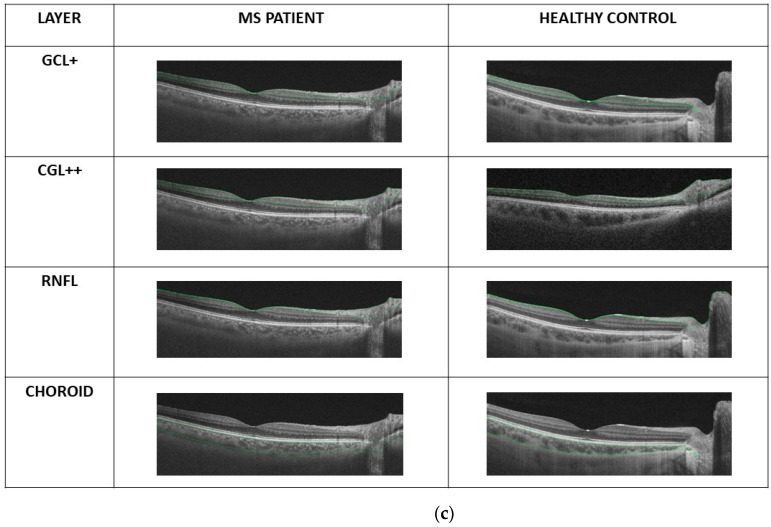
(**a**) Retinal layer measurements analyzed: RNFL, GCL+, GCL++, complete retina and choroid; (**b**) OCT scanning source slice image of a normal eye showing, in green, the boundaries of the layers into which the software segments the neuroretina image and the representation of the complexes measured; (**c**) representation of delimitation of the four retinal layers determined by the segmentation software of Triton OCT (optical coherence tomography) in a patient with multiple sclerosis and in a control subject: GCL+ (ganglion cell layer +: between the boundaries of the retinal nerve fiber layer and the inner nuclear layer, therefore including the GCL and the inner plexiform layer), GCL++ (between the boundaries of the inner limiting membrane and the inner nuclear layer, therefore including the retinal nerve fiber layer and the GCL+), RNFL (retinal nerve fiber layer: between the boundaries of the inner limiting membrane and the GCL) and CHOROID (from Bruch’s membrane to the choroidal-scleral interface).

Measurements were obtained for the following structures (Figure 1): whole retina (measured between the boundaries of the inner limiting membrane (ILM) and the retinal pigment epithelium (RPE)); retinal nerve fiber layer (RNFL) (between the boundaries of the ILM and the ganglion cell layer (GCL)); GCL+ (between the boundaries of the RNFL and the inner nuclear layer, therefore including the GCL and the inner plexiform layer); GCL++ (between the boundaries of the ILM and the inner nuclear layer, therefore including the RNFL and the GCL+). The choroidal (posterior vascular structure) thickness was measured from Bruch’s membrane to the choroidal–scleral interface.

The OCT device is regularly serviced by the manufacturer (Topcon) as per its proprietary protocols. The device provides a quality scale (range 0–100) to indicate the signal strength index (SSI), where SSI = 0 indicates poor quality and SSI = 100 indicates excellent quality. In our study, only images considered “good quality” were analyzed (SSI > 55). All scans were obtained by a single operator with extensive experience of performing the OCT technique who was blinded to the group classification.

Scans were evaluated by the operator to test that they fulfilled the human-led validated consensus quality control criteria (OSCAR-IB) [68], for OCT and the criteria embedded into OCT reporting guidelines (APOSTEL) [69].

Built-in DRI-OCT software (v 10.1.3.43469) was used to segment layers and construct topographic maps using the thicknesses of each of the structures analyzed.

### 2.3. OCT Map Processing

**Figure 2:**
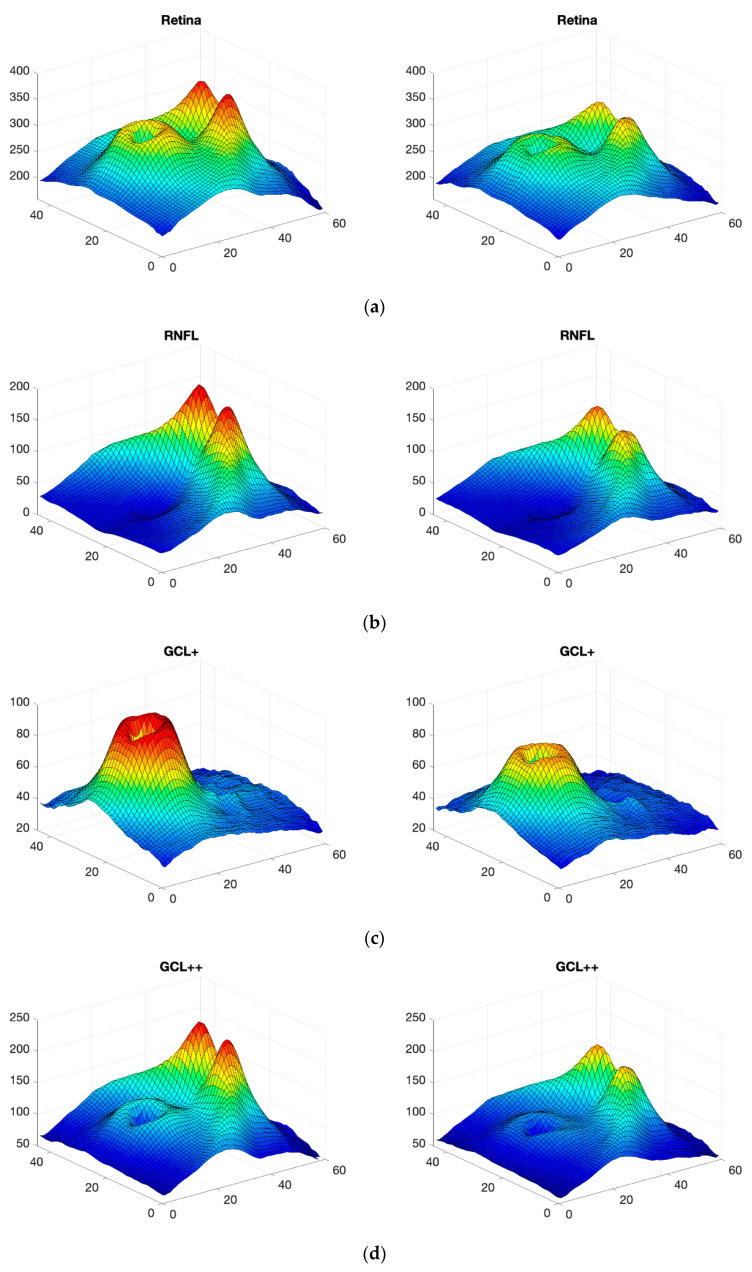

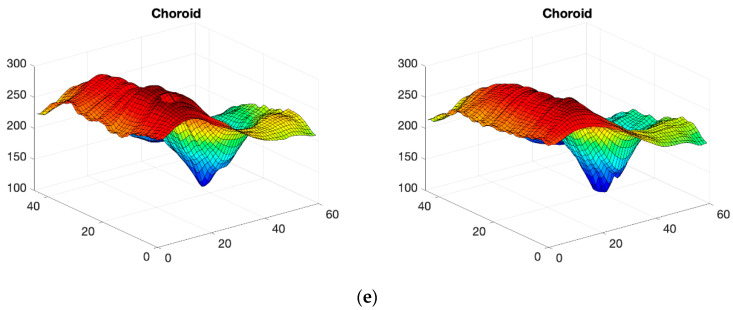
3D images of the 5 structures obtained with OCT in real subjects; mean value in all control subjects (left) and mean value in MS patients (right). (**a**) complete retina; (**b**) RNFL; (**c**) GCL+; (**d**) GCL++; (**e**) choroid.

The information on the thicknesses of the 5 structures (Figure 2) is pre-processed before it is applied to the CNN. The intention in doing so is to identify the information with greatest discriminant capacity in order to use it in performing diagnosis on control subjects and MS patients.

#### Thickness Image Pre-Processing

The discriminant capacity of the images is identified using effect sizes (Cohen’s d) [70]. For each of the 5 structure thicknesses available, the mean value and the standard deviation in each pixel (x,y) in the control subject ($M_{\mathrm{CR}}\left(x,y\right),{\;\mathrm{SD}}_{\mathrm{CR}}\left(x,y\right)$) and patient ($M_{\mathrm{MS}}\left(x,y\right),{\;\mathrm{SD}}_{\mathrm{MS}}\left(x,y\right)$) images, respectively, is calculated. Cohen’s d is the difference between two means expressed in standard deviations:

(1) dL(x,y)=MCRL(x,y)−MMSL(x,y)[ (NCR−1)SDCR2(x,y)+(NMS−1)SDMS2(x,y)NCR+NMS−2 ]−12  x=(1,…45) y=(1,…60)

Superscript L refers to one of the 5 structures (complete retina, RNFL, GCL+, GCL++, choroid) and N_CR_ and N_MS_ refer to the number of control subjects and patients, respectively. According to Cohen’s criterion, d values > 0.8 indicate a large effect. In our case, d ≥ 1.02 is necessary to ensure good discriminant capacity; this threshold obtains the maximum area under the curve in mean value for all the layers and measurement points. The same threshold is used for all layers.

The value of Cohen’s parameter is calculated for all the pixels (x,y) in the 5 layers. For those that exceed the threshold set, the thickness value is maintained. Conversely, those pixels that do not exceed the threshold are assigned a value of 0:

if

(2) $$d^{L}\left(x,y\right)\geq 1.02\;:{\;p}^{L}\left(x,y\right)={\;p}^{L}\left(x,y\right);$$

or else:

$$p^{L}\left(x,y\right)=0;$$

Thus, the images used as inputs to the CNN ($p^{L}\left(x,y\right)$) only include information on the pixels that contain data relevant to diagnosis of MS.

### 2.4. CNN Architecture

**Figure 3:**
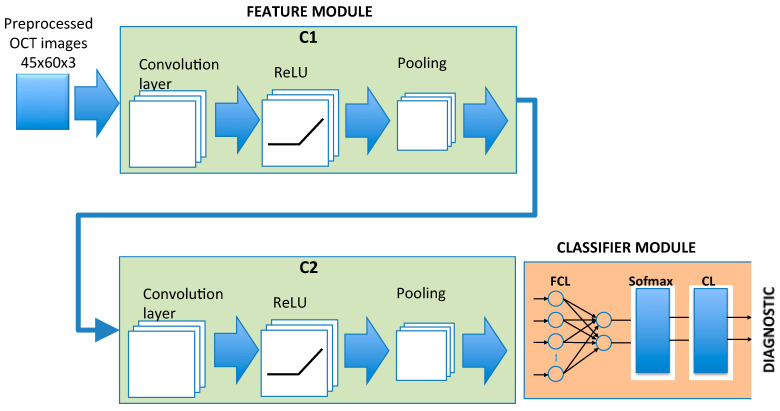
CNN architecture implemented. **C1**, **C2**: convolutional submodules. FCL: fully connected layer. CL: classification layer.

Taking into account the size of our database, a CNN has been designed with a lower number of adjustable parameters than other state-of-the-art architectures, such as DenseNet (>0.8 M), GoogleNet (7 M), ResNet18 (11.7 M), Inception V3 (29.3 M), VGG-16 (138 M), or AlexNet (60 M), thus reducing both the chances of overfitting and training times. Figure 3 shows the overall structure of the CNN implemented. The feature module is implemented with 2 cascaded convolutional submodules (C1, C2), each of them including convolution, nonlinear transformation and sublayer pooling. The input images have the dimensions height (h), width (w) and number of structures or neuroretinal layers analyzed (L).

Convolution of the input images is performed in submodule C1 and a nonlinear function is applied to the outcome, reducing the amount of data. Performing convolution (dot product) between the input images and N_F1_ filters or kernels produces N_F1_ feature maps. The dimensions of each feature map depend on the original dimensions of the images and the stride and zero-padding parameters defined in the convolution operation. The stride or step size (s_1_,s_1_) is the number of pixels by which the kernel shifts over the input image in each step and determines the overlap between individual output pixels. At the output of each convolutional sublayer a padding (p_1_,p_1_) of zeros is added to all the edges of the output of each feature map.

The next layer within C1 is a nonlinear transformation of the results of convolution, modifying the values of the feature maps in order to improve training convergence. Functions commonly used include: ReLu (f(x)=max(0,x), sigmoide, tanh, sofplus: $f\left(x\right)=\ln\left(1+e^{x}\right)$, etc.

The final layer is the subsampling or pooling operation, the purpose of which is to reduce the dimensions of the feature maps. Each feature map is divided into regions of N_H_*N_W_ dimensions and the values in this window are summarized in one pixel, generally the maximum (MaxPooling: the strongest activations over a neighborhood are prioritized) or mean values (MeanPooling).

The second submodule (C2) shares a similar structure to C1: number of filters (N_F2_), stride (s_2_,s_2_), padding (p_2_,p_2_), nonlinear transformation and pooling. Its inputs, however, are the N_F1_ feature maps at the output of C1.

The output of C2 is the input to the classification module comprising a fully connected layer (FCL), implemented with a feed-forward neural network (FNN). The FNN is structured into two layers: the input layer and the output layer (2 neurons; classes in the dataset: MS or control) and all the input neurons are connected to the two output neurons and assigned a weight and an adjustable bias. The FCL outputs are normalized using a softmax function that assigns probabilities to the output of the FCL according to:

(3) $$p\left(i\right)=\frac{e^{z_{i}}}{\sum_{j}e^{z_{j}}}\;$$

where *i* represents the class (control, MS) and *z_j_* the outputs of the fully connected layer.

The classification layer (CL) uses the probabilities returned by the softmax activation function for each input to assign the input to one of the mutually exclusive classes.

The CNN’s structure is defined in detail below, taking into account that the three neuroretinal structures analyzed are those with greatest discriminant capacity: complete retina, GCL+ and GCL++ (see Section 3). The CNN was implemented in Matlab using the Deep Learning Toolbox. All the training and testing were carried out on a PC (Intel Core i7-9700, 32 GB) with a Nvidia GeForce RTX 2070 GPU.

The input dimensions of the CNN are (h,w,L) = (45,60,3). The best diagnostic results were obtained by convoluting the images with 64 filters in C1 (N_F1_ = 64) with dimensions (d_1_,d_1_) = (7,7), stride (s_1_,s_1_) = (1,1) and padding (p_1_,p_1_) = (0,0). Therefore, in the C1 convolution sublayer, 64 feature maps are obtained.

For the nonlinear transformation of the convolution values, and in order to improve the training, the ReLu function is used: σ(x)=max(0,x). This operation does not change either the number of feature maps or their dimensions. In the C1 polling layer, dimension windows (N_W_,N_W_) = (2,2) with offset (S_W_,S_W_) = (2,2) are defined. There is therefore no overlap between the windows, and in each window, the pixel with the highest value is selected (MaxPooling). In short, the output of C1 consists of 64 feature maps with dimensions (h_POOLING1_,w_POOLING1_) = (19,27).

Submodule C2 has the same characteristics as C1. Therefore, the input to the classification module has 64 feature maps with dimensions (h_POOLING2_,w_POOLING2_) = (6,10).

In this condition, the number of inputs to the full connected layer is 3840 (6 × 10 × 64 + 2 bias). The FCL multiplies the input by a weight matrix and then adds a bias vector. This output is connected to the softmax layer, which applies a softmax function to the inputs (normalizes the input vector and obtains a probability distribution). Finally, a classification layer computes the cross-entropy loss for multi-class classification problems with mutually exclusive classes. In our network, we defined 2 outputs (“MS” and “control”).

### 2.5. Training of the CNN

The training process using the image dataset applies the leave-one-out cross-validation method (LOOCV), which is a model validation technique for assessing how the results of a statistical analysis will generalize to an independent dataset. Considering that data are available for 48 control subject eyes and 48 MS patient eyes, the process implemented to evaluate the CNN’s performance is as follows:

1. An eye of a control subject that will not be used in training neither is selected. The remaining 47 control subject eyes are used to train a GAN (Section 2.6) to generate n = 100 synthetic control images, while the 48 MS patient eyes are used to train another GAN to generate n = 100 synthetic MS images. The process is performed on the complete retina, GCL+ and GCL++.
2. The Cohen thresholding described above is applied to the total number of images available for each of the 3 layers (147 control eyes, 148 MS eyes), which are used to train the CNN.
3. The trained CNN is tested on the images of the eye that was not used either to generate the synthetic images or to train the CNN. The result of the classification is taken into account with regard to the data in the confusion matrix.
4. Points 1–3 are repeated until all the control eyes have been tested.
5. Points 1–4 are repeated, but in this case leaving out, one by one, all the MS patient eyes.

The GAN method increases the size of the database used to train the CNN. By implementing the leave-one-out procedure, the CNN test is performed with data not used in training the CNN in either the data augmentation phase or as part of the training set. Performing this process for all possible folds produces the corresponding confusion matrix.

In each of the N (48 + 48) training steps, the error (E(k)) between the CNN output and the correct response is evaluated with the cross-entropy loss function [71]. The network parameters (weights and biases) are adjusted by applying the Adam (Adaptive Moment estimation) optimization method [72]. Adam uses estimations of first and second moments of gradient to provide adaptive learning rates. The first (m: exponentially decaying average of past gradients) and second moments (v: exponentially decaying average of past squared gradients) are defined as:

(4) $$m\left(k\right)=\beta_{1}m\left(k-1\right)+\left(1-\beta_{1}\right)\frac{\Delta E}{\Delta w}\;$$

(5) $$v\left(k\right)=\beta_{2}v\left(k-1\right)+\left(1-\beta_{2}\right)\;{\left(\frac{\Delta E}{\Delta w}\right)}^{2}\;$$

*β*_1_ and *β*_2_ are decay rates for the first and second moments, respectively. As the previous values of *m*(*k*) and *v*(*k*) may be biased towards zero, they are corrected with bias-corrected moment estimates:

(6) $$\hat{m\left(k\right)}=\frac{m\left(k\right)}{1-\beta_{1}}\;$$

(7) $$\hat{v\left(k\right)}=\frac{v\left(k\right)}{1-\beta_{2}}$$

The CNN’s adjustable parameters are updated according to the following:

(8) $$w\left(k+1\right)=w\left(k\right)-\frac{\alpha .\hat{m}\left(k\right)}{\sqrt{\hat{v}\left(k\right)}+\varepsilon }\;$$

The hyperparameters used to train the CNN are $\alpha =0.0001,\;\beta_{1}=0.9,\;\beta_{2}=0.999,\;\epsilon =10^{-8}$ and maximum number of epochs (=1000).

### 2.6. OCT Data Augmentation

**Figure 4:**
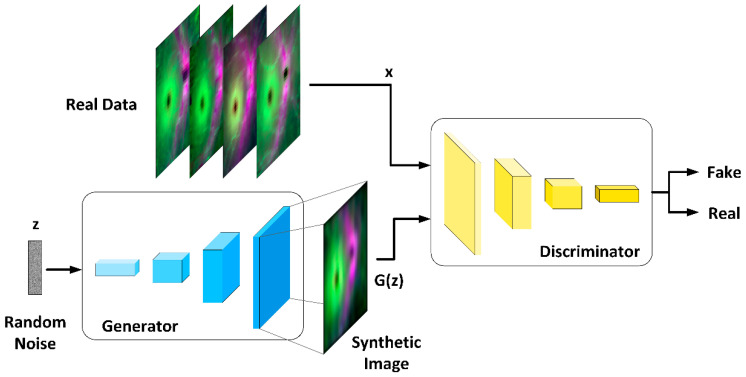
GAN framework workflow.

Our goal is to augment the set of images of thicknesses of the retinal layers with greatest discriminant capacity in order to increase the size of the CNN training set. Figure 4 illustrates the workflow of the basic GAN framework. The GAN architecture is implemented as a two-neural-network system, where the two networks are alternately trained, competing in a zero-sum approach. The neural network (G: generator) produces fake (G(z) data from a random noise (z) input with distribution p(z) (generally Gaussian or uniform).

The discriminator (D) is trained to maximize the probability of classifying both real and generated data as real images. G is trained to minimize log(1−D(G(z)), the divergence between the two data distributions (maximally confuses the discriminator).

The training goal of the GAN model is to maximize the output of discriminator D and minimize the output of generator G:

(9) $$\underset{G}{\min}\;\underset{D}{\max}\;L\left(D,G\right)={\;Ε}_{x}[\log\left(D\left(x\right)\right]+{Ε}_{z}[\log\left(1-D\left(G\left(z\right)\right)\right]\;$$

where Ε signifies expectation, **x** (OCT images) denotes the real input data, **z** (unidimensional random noise array) denotes the noise input into the generator, and G(**z**) is the data generated by the generator. D(**x**) in a scalar that indicates the probability that D judges that x comes from the real-image distribution. D(G(z)) is the discriminator’s estimate of the probability that a fake image (G(z)) is real. D(G(**z**)) is the probability that the discriminator will judge whether the data distribution generated by the generator is real or not.

To resolve the stability issues and meet the need for a lot of training skills of the original GAN (which used fully connected neural networks: multilayer perceptron), we used the Deep Convolutional Generative Adversarial Network (DCGAN) proposed in [73]. In DCGAN, the generator and the discriminator are implemented with CNNs, albeit with different architectures, as described below.

#### 2.6.1. Generator Architecture

**Figure 5:**
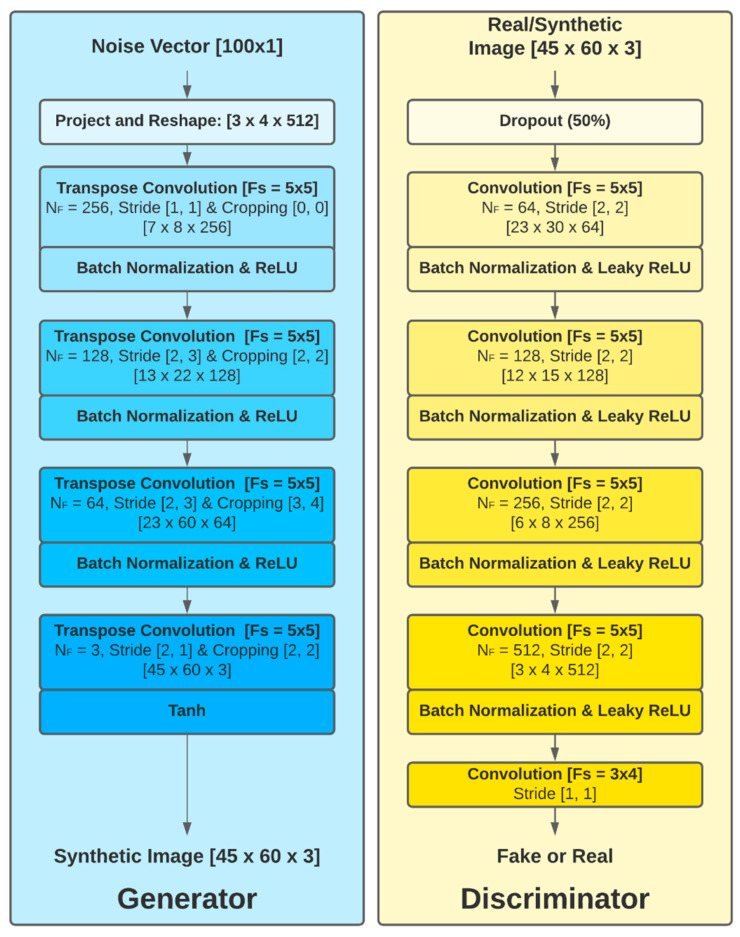
Generator and discriminator architecture. NF: number of filters; Fs = filter dimensions.

The architecture of the generator (G) is based on the model proposed in [73] (Figure 5). G is a deep inverse convolution network, with the input comprising a unidimensional random noise array (100 × 1) following a standard normal distribution (μ=0, σ=1), mapping it onto the G(**z**) output (dimensions 45 × 60 × 3). The G network is comprised of a project and reshape layer followed by four transposed convolutional layers and ending with a hyperbolic tangent activation function.

The project and reshape layer maps the random vector **z** of size 100 × 1 onto a 3 × 4 × 512 array through a linear, fully connected layer. The transpose convolution operation is typically used to upsample the feature space map to a desired output applying learnable parameters [74]. The number of filters (size 5 × 5) is decreased progressively from 512 on the first layer to 3 on the last one, matching the dimensions of the expected synthetic image. In each layer, the stride and cropping configuration of the transposed convolution is adapted in order to obtain a final output image of dimensions 45 × 60 × 3. Batch normalization and ReLU activation are implemented on the output of each convolution layer except the last one, on which normalization is not performed and a hyperbolic tangent activation function is used. Batch normalization is a common technique that normalizes the layer’s outputs by re-centering and re-scaling the data so as to obtain a stable solution more efficiently [75].

The following describes in detail the process by which an input with dimensions [100 × 1] is converted into images with dimensions [45 × 60 × 3]. A unidimensional vector [100 × 1] is passed through a project and reshape layer, upscaling the input to a [3 × 4 × 512] tensor. After reshaping the noise vector and passing through the first transpose convolution layer, the dimensions change from [3 × 4 × 512] to [7 × 8 × 256]. The batch normalization (BN) layer and ReLU layer do not change the data dimensions. Then, passing through the second transpose convolutional layer, the dimensions are modified from [7 × 8 × 256] to [13 × 22 × 128]. Replicating the structure presented before, the data pass through the same BN layer and ReLU layer. In the third stage, the dimensions change from [13 × 22 × 128] to [23 × 60 × 64] and, in the last transpose convolution, the dimensions of the data are changed from [23 × 60 × 64] to the desired output size [45 × 60 × 3]. Finally, the data pass through the tanh layer to obtain the synthetic OCT image.

#### 2.6.2. Discriminator Architecture

The discriminator is an approximate mirror of the generator, but with the difference that convolutional layers are used to reduce the dimensions of the input images to a binary output; essentially, a CNN configured as per the guidelines set out in [73] is implemented. The inputs of the D network are the real and synthetic images with dimensions [45 × 60 × 3]; each of the channels includes the thickness data of the complete retina, GCL+ and GCL++.

Dropout regularization (50%) is applied, which prevents all the neurons in the next layer from converging to the same goal, decorrelating the weights. Next, 5 convolutional layers with an increasing number of filters (size = 5 × 5) on each layer are applied. The Leaky ReLU (f(x)=max(0,x)+0.002.min(0,x)) activation function is used for all the layers. The process of scaling the data across the five convolutional layers to the output is [45 × 60 × 3] → [23 × 30 × 64] → [12 × 15 × 128] → [6 × 8 × 256] → [3 × 4 × 512] → [1]. The output of the discriminator is the probability of the input image belonging to the real sample image.

#### 2.6.3. DCGAN Training

The DCGAN architecture described above is used to generate synthetic OCT images [45 × 60 × 3] for control subjects and MS patients. As three retina layers were found to have the greatest discriminant capacity based on thickness values (complete retina, GCL+, GCL++), for each eye, and to maintain the correlation between them, a three-channel image is created.

The two networks were trained using a mini-batch size of 32 for 500 epochs. The Adam optimization method [72] was used with a learning rate of α=0.0002, a gradient decay factor of $\;\beta_{1}=0.5$ and a square gradient decay factor of $\beta_{2}=0.999\;\mathrm{and}\;\epsilon =10^{-8}.$

Training is performed to generate synthetic images of control eyes, leaving out the data of one of the subjects (leave-one-out); once finished, 100 random **z** vectors are generated and 100 synthetic control images are obtained at the output of the generator. The same process is repeated to generate synthetic OCT images for MS patients. Both types of synthetic image will serve to augment the main CNN training set.

## 3. Results

### 3.1. Database

In order to build an effective diagnostic tool, the sample size needed to detect differences of at least 5 μm in GCL+ thicknesses measured by Triton OCT—applying a bilateral test with α = 5% risk, β = 10% risk (i.e., with a power of 90%), and an unexposed/exposed ratio of 0.5—amounts to at least 86 eyes (43 from healthy subjects and 43 from MS patients) [76]. The database is made up of OCT images taken from N_MS_ = 48 MS patients (male/female: 9/39; age 43.79 ± 8.41 years) and N_CR_ = 48 control subjects (male/female: 10:38; age 44.44 ± 7.18 years). There is no significant difference in mean age between the two groups (*p* = 0.107, Student’s *t*-test) or in the distribution between sexes (*p* = 0.451, χ2-test). The patients have recently been diagnosed (mean ± standard deviation: 7.35 ± 1.95 months) and their EDSS score (median [interquartile range]) is 1.07 [0.35]. A value of EDSS = 1.0 means that the patients have no disability (minimal signs in one functional system).

One eye from each patient is randomly selected for inclusion in the analysis. The only exception is if one of the patient’s eyes does not meet the inclusion criteria. In that case, the eye that meets the criteria is selected.

### 3.2. OCT Image Pre-Processing

**Figure 6:**
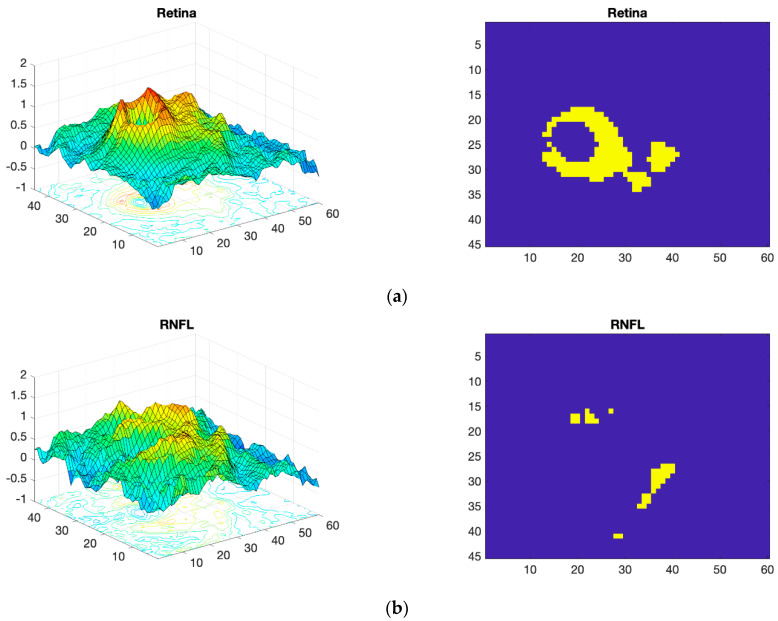

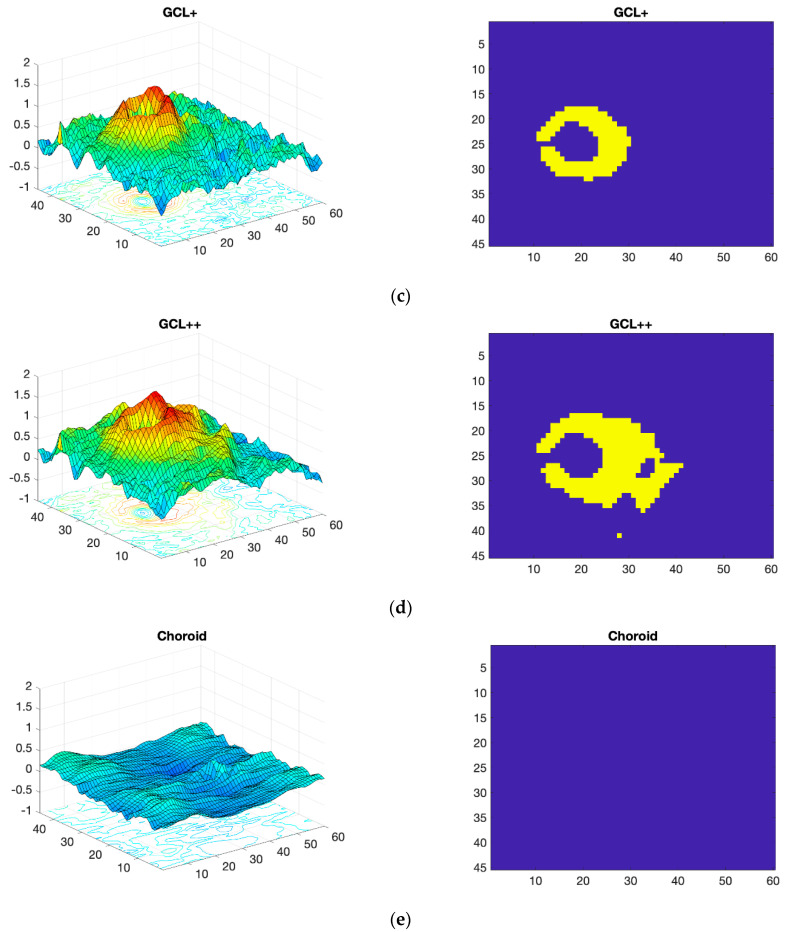
Processed OCT images of real subjects. Left: Cohen’s d value for the various structures. Right: the best regions, selected with a threshold of d_TH_ = 1.02 (identical for all layers), are shown in yellow. (**a**) Complete retina; (**b**) RNFL; (**c**) GCL+; (**d**) GCL++; (**e**) choroid.

In the first step, the d value of the five layers is calculated. Figure 6 (left) shows the d values obtained with Equation 1 for the different neuroretinal structures. As can be seen, the RNFL and choroid layer show a very uniform range of d values. In contrast, the complete retina, GCL+ and GCL++ present a wide range of d values. Later, all layers are thresholded using a fixed threshold. In this case, a d_TH_ threshold of 1.02 (identical for all layers analyzed) has been used. Figure 6 (right) shows the best areas in each of the structures of the retina. Analysis of the results in Figure 6 reveals that the RNFL and choroid barely show alterations in thickness due to the presence of MS. These structures are therefore discarded from automatic diagnosis.

In the complete retina, and in GCL+ and GCL++, the topographical distribution of the most discriminant pixels are very similar, presenting a circular grouping around the macula (horseshoe-like shape or U zone) [44], weakening the discriminant capacity in the temporal macular area. According to the criterion set, the layer that provides most information is the GCL++ (44.99% of the 45 × 60 points exceed the threshold and are therefore considered discriminant), followed by the complete retina (26.71% considered discriminant) and the GCL+ (22.93% considered discriminant).

Consequently, the information input into the CNN will be the images corresponding to the GCL++, complete retina and GCL+, processed according to (2).

### 3.3. Data Augmentation

The data augmentation process for the control subject and MS patient OCT images is performed for subsequent use in training the CNN. As the retinal layers with greatest capacity to discriminate between control subjects and MS patients are the complete retina, GCL+ and GCL++, 100 control subject images and 100 MS patient images are synthesized for each of these layers.

**Figure 7:**
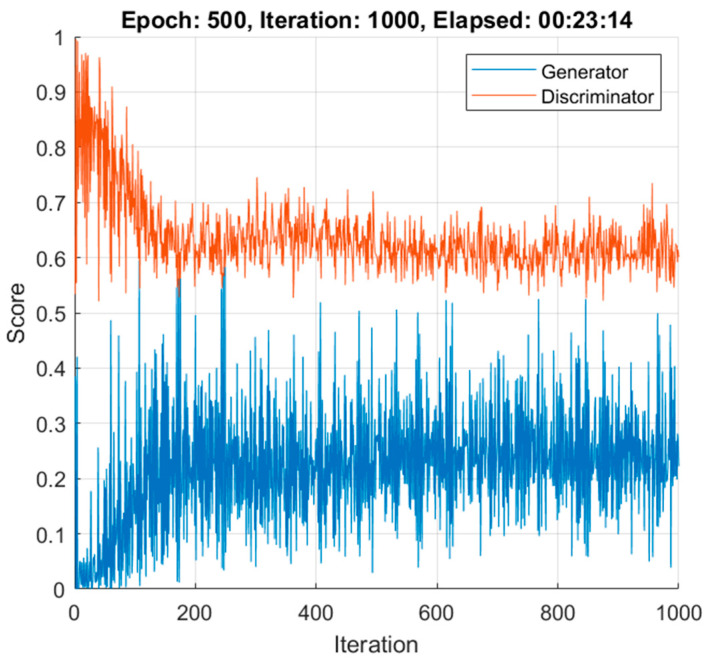
Generator and discriminator learning curve loss over time.

**Figure 8:**
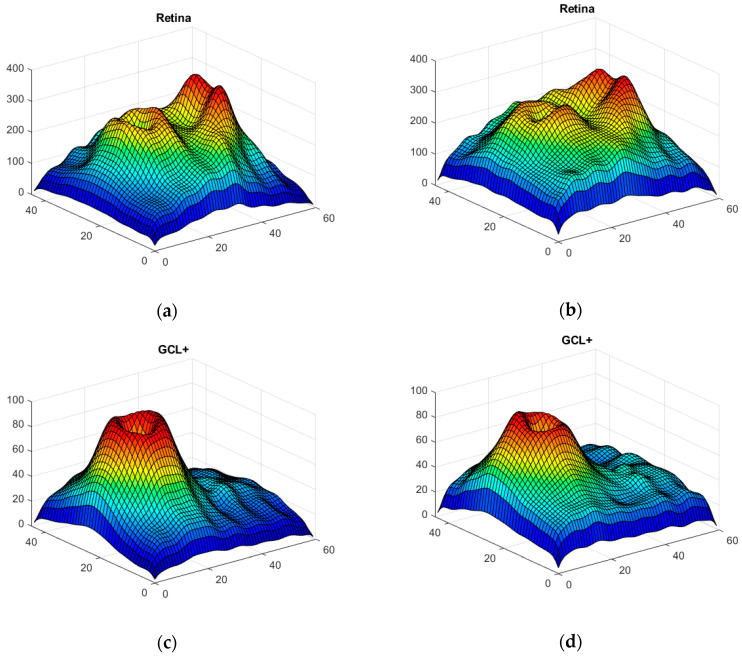

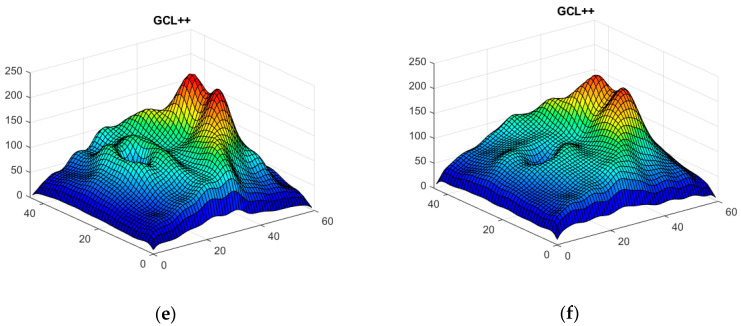
3D images of the 3 structures synthesized with DCGAN; mean value in all control subjects (left) and mean value in MS patients (right). (**a**,**b**) Complete retina; (**c**,**d**) GCL+ layer; (**e**,**f**) GCL++ layer.

Figure 7 shows the generator and discriminator losses over iterations. In order to verify the accuracy and reliability of the GAN method, Figure 8 shows the mean value for the 100 synthetic images (the case shown corresponds to when the data of the first control subject have not been used, as per the procedure described in Section 2.4), both for control subjects and MS patients (the 48 available images have been used as real data); comparing them with the real images (Figure 2) reveals that the outcome of the data augmentation process is satisfactory.

### 3.4. Classification Results

To obtain the confusion matrix as per the procedure described in Section 2.5, it is necessary to test the CNN over 48 + 48 cycles, training the CNN each time. In each cycle in which a control subject is tested, the MS patients’ data augmentation set (real data = 48 patients) is not modified; consequently, the GAN process is applied 48 times to obtain synthetic images of control subjects (using 47 real images) and 1 time to obtain synthetic images of MS patients (in the GAN process, 48 real images are used). The computational load is the same in the cycles in which the CNN test is performed with real MS patient images.

Tests were performed to evaluate the effect of the number of synthetic images generated, using the same *n* value for patient images and control images. The values tested were *n* = 25, *n* = 50, *n* = 75, *n* = 100, *n* = 125, and *n* = 150; it was found that the best results were obtained for *n* ≥ 100.

The final result of the CNN test is shown in Table 1, for *n* = 100. It shows the confusion matrix, which displays 100% success in classifying the database available. Therefore, sensitivity = specificity = 1.

As shown in the confusion matrix, our method is successful in predicting which eyes belong to healthy subjects and which eyes belong to MS patients in all cases in our population. Although further studies would be necessary to validate the results in other populations, our findings show that this classification method has the highest positive and negative predictive value in diagnosing early-stage MS through the study of the optic nerve.

### 3.5. Running Time Evaluation

Using a personal computer (Intel Core i7-9700, 32 GB) with an Nvidia GeForce RTX 2070 GPU, the following run times were obtained:

- Generation of *n* = 100 synthetic patient images + 100 synthetic control images: 23 min.
- CNN training (147 control images, 148 patient images): 25 min.
- Testing of a single control subject’s images: 0.2 s.

The method is therefore well-suited to clinical settings due to its extremely short run times, especially in the decision-making (test) phase.

## 4. Discussion

This paper proposes and implements a deep-learning approach to the automatic diagnosis of early-stage MS by analyzing structural neuroretinal data obtained using SS-OCT. The main contributions are considered to be (i) a very efficient and accurate method of diagnosing early-stage MS using a CNN, and (ii) effective implementation of OCT image data augmentation to improve CNN training.

Very few papers have investigated the MS diagnosis potential of analyzing retinal thickness data using artificial intelligence. By combining clinical data and RNFL thicknesses, Montolío et al. [49] obtained an accuracy of 87.7% with an ensemble classifier. This, however, is achieved in patients with a prolonged disease duration (mean of 10.15 ± 8.30 years since diagnosis of MS); therefore, in these patients, there is a significant loss of RNFL thickness associated with axonal deterioration.

Early-stage MS has a subclinical structural [30] and electrophysiological [77,78] effect on the retina, possibly as a consequence of retrograde and anterograde axonal degeneration and microglial involvement, among other factors. This damage is only detectable with state-of-the-art, high-resolution tests such as spectral-domain or swept-source OCT and is imperceptible to the human eye during examination of the fundus. Previous studies of newly diagnosed patients have shown that alterations in the thicknesses of the different layers of the retina detectable with this OCT technology can be used to diagnose MS as early as the first few months of development of the disease. Using data from the same cohort of control subjects and patients, advanced summaries have been published regarding the advantages of combining retinal thickness data with signal-processing techniques in the diagnosis of MS.

The database used in this study has been employed in two prior studies (Table 2). The first [30] uses the average thicknesses of certain regions of the retina (for each layer of the retina, thicknesses of nine macular areas, four quadrants and six sectors of the peripapillary area are obtained). The three most discriminant features are then selected and applied to an SVM, obtaining an accuracy value = 0.91 in diagnosis. The second paper [31] uses OCT images of the same subjects (controls and patients) but applies the Wide protocol. This protocol makes it possible to explore the neuroretina with greater specificity, point-to-point, without depending on mean values in certain regions, and enables the identification of the most discriminant layers and zones using the Cohen distance (Figure 6), particularly in the papillomacular bundle (U-zone). Identification of the most discriminant measurement points is performed using the Cohen distance, following the same method described in this paper, and their thickness values constitute the feature vector. Several classifiers (SVM and classic feed forward neural network (FFNN)) were tested, the best result (accuracy = 0.97) being obtained with an FFNN model with 10 neurons in the hidden layer.

To our knowledge, this paper is the first to use a CNN to analyze retinal thicknesses to diagnose MS. One of the advantages of CNNs is that they can be considered a black box in which correspondences are established between inputs and outputs, and in many cases, explicit feature extraction is not necessary. However, in the approach adopted in this paper, the features were pre-selected, considering as inputs to the CNN the retinal structures that were most affected by the disease and filtering out the pixels with little discriminant capacity. Under these conditions, and using our database, the CNN is capable of learning the mapping between OCT images and the diagnoses of recent MS patients, producing flawless classification (sensitivity = specificity = 1).

Since successful training of deep network architectures requires the availability of large and balanced datasets, control subject and MS patient OCT image data augmentation was performed. In this case, the GAN system modules/networks were implemented using a CNN, as per [73].

The results of the data augmentation implemented are fully satisfactory, as evidenced both by the images generated (Figure 8) and the perfect classification produced by the CNN; tests were performed varying the number of synthetic images (*n* = 25, 50, 75, 100, 125 and 150), with the best results being obtained from *n* = 100 upwards. It should be noted that the CNN test was performed using LOOCV, ensuring that each test image had not been used in either the data augmentation phase or the CNN training phase.

The findings of our study suggest that it is possible to detect neuroretinal structural alterations using CNNs in the initial phases of the disease and at an excellent level of reliability. However, in medicine, we consider that any new algorithm or tool needs to be validated with a broader, multicenter database of control subject and MS patient OCT images, preferably taken from other populations subject to different conditioning genetic and environmental factors. If the size of the available database is adequate in terms of the number of real images available, and if it is suitably balanced, it may not be necessary to implement data augmentation before training the CNN.

We also consider it worthwhile to research the capacity of artificial intelligence to diagnose MS and its potential development, taking into account other patient variables that could supplement diagnosis, such as MRI, electrophysiological signals (principally visual-evoked potentials) [77] and clinical data.

It is difficult for neurologists to predict the course of MS in patients as the pathology is extremely variable and unpredictable. In some cases, the disease develops rapidly in the first few years before later stabilizing, leading to a decrease in the number and intensity of outbreaks, while in others, the aggressiveness of the disease increases with time. There is no reliable tool with which to determine how the disease will develop in each patient and, in this sense, finding tools that facilitate personalized medicine would be highly beneficial as they could indicate which drug to apply in each case. In a pathology such as MS, characterized by constant changes in medication, most of which entails high costs for health systems, having a method capable of detecting patients in whom more aggressive development or greater neurodegeneration are expected would allow practitioners to select the most efficient drugs for these patients (this medication usually being the most expensive or presenting the most side effects).

Therefore, one of the objectives of researchers focusing on neurodegenerative diseases and on the utilization of neuroimaging tests in diagnosis, monitoring and prognosis is the use of biomarkers that, identified in noninvasive tests, can be simply applied in daily clinical practice. OCT can be easily implemented for this purpose since it is a noninvasive test that can be performed by staff without specific training in a matter of minutes and without causing patient discomfort. Furthermore, it is cost-effective; testing all patients with these pathologies is relatively viable since most medium-sized hospitals, and even numerous health centers, already have this technology, meaning that the method does not require the acquisition of additional devices.

Biomarkers for MS can help diagnose the disease, predict its course, or determine the outcome of treatment. As yet, there is no single accurate, reliable diagnostic test [79].

The usefulness of OCT as a noninvasive tool in the diagnosis and follow-up of MS is increasingly accepted by researchers. Some researchers even suggest that its usefulness is similar to that of magnetic resonance imaging, as it allows in vivo visualization of the state of neurodegeneration at the axonal level and can be repeated as many times as necessary as it is a completely innocuous technique that does not irradiate the patient’s brain and does not cause any discomfort, except for that induced by looking at a fixed point for a few seconds [80,81].

Neuroimaging techniques such as magnetic resonance imaging are expensive, very time-consuming, and require several people to carry them out. In contrast, OCT eye-imaging devices are widely available and the technique can be performed by any professional as it has a short learning curve, takes up little space and does not require compliance with particular technical specifications or the construction of lead walls in the room in which it is installed. It is usually available in most health centers and hospitals with an ophthalmology service, making it a very cost-effective test as no additional investment other than that involved in ensuring coordination and collaboration between the ophthalmology and neurology services is required to deploy it [82].

The new algorithms proposed in this paper, once they have been validated in other populations, could be implemented in commercially available OCT devices following simple modification of their software. They would thus constitute an important advance entailing little investment or expense for health systems.

This study has several limitations. The principal one is that no differential analysis was conducted between MS treatment groups. Another limitation is that decision making is only made on the basis of OCT data; the performance of the classifier on multicenter and larger databases could probably be improved by using other clinical data, such as age, the presence of signs or symptoms in the patient, gender, etc. Another limitation of this study is that it is only cross-sectional; a longitudinal study could be conducted to analyze the usefulness of the tools as methods of monitoring disease progression, treatment efficacy or even prognostic capacity to detect those subjects at greatest risk of aggressive development of the disease.

Based on our findings, the implementation of the new algorithms in most large hospitals that have neurology and ophthalmology services appears to be completely viable. Essentially, it only requires collaboration between the two specialties to coordinate the visits and interpret the results. At the same time, OCT is an easily interpreted test that could be evaluated directly by a neurologist with minimal training. If our proposal works sufficiently well in other populations, it would be feasible to implement it in the software of devices already on the market so that when the test is performed on a patient diagnosed with a suspected neurodegenerative pathology, the device would efficiently predict the probability of the patient presenting that pathology.

This tool meets the objective of providing personalized medicine, which is considered to be the immediate future of healthcare by most researchers in this area.

## 5. Conclusions

The method presented in this paper represents an advance in the diagnosis of early-stage MS using data on retinal thicknesses analyzed using artificial intelligence. We believe that this method can also be used to predict disease progression in other disorders that affect the central nervous system (Alzheimer’s, bipolar disorder, etc.), or to diagnose them. In any case, it is advisable to examine other data of interest, such as age, the presence of clinical symptoms or alterations in brain neuroimaging, in addition to retinal thicknesses, in order to draw robust conclusions.

Effective configuration of the neural networks—both in the classifier and the GAN—was achieved by adopting the trial-and-error strategy, starting with the simplest structure (one convolutional layer) and in each phase increasing the number of layers. In each phase, testing of parameters such as number of filters, size, etc. was also performed by trial and error based on exhaustive search. The search for optimal architectures was automated using a program that analyzed a wide range of possible options. In future papers, it would be desirable to implement more advanced automated neural architecture search strategies [83]. Future directions for our line of research could include the validation of these tools in external populations of other ethnicities and from other geographical locations. We could also work on improving these diagnostic algorithms by incorporating other clinical parameters (e.g., age, sex, presence or absence of certain signs or symptoms, etc.). More longitudinal studies with larger numbers of patients are needed to clarify the new tools’ usefulness in assessing disease progression, disease monitoring and treatment effectiveness.

If research proves effective, a diagnostic tool based on relatively affordable equipment commonly found in hospitals and augmented with inbuilt real-time diagnostic software would be achievable.

## Figures and Tables

**Table 1:** Confusion matrix. TN: true negative, FP: false positive, FN: false negative, TP: true positive.

	Actual MS	Actual Control
Predict MS	TP = 48	FP = 0
Predict control	FN = 0	TN = 48

**Table 2:** Comparison of the results of several methods using the same OCT database. TN: true negative, FP: false positive, FN: false negative, TP: true positive, FFNN: feedforward neural network, SVM: support vector machine.

Method	Confusion Matrix Results				
	TN	FP	FN	TP	Accuracy
Average thicknesses. Gaussian SVM [30]	44	5	43	4	0.90
Wide protocol. Cohen’s d. Linear SVM Classifier [31]	41	7	7	41	0.85
Wide protocol. Cohen’s d. Quadratic SVM Classifier [31]	40	8	6	42	0.83
Wide protocol. Cohen’s d. Cubic SVM Classifier [31]	38	10	5	43	0.79
Wide protocol. Cohen’s d. Fine Gaussian SVM Classifier [31]	43	5	29	19	0.89
Wide protocol. Cohen’s d. Medium Gaussian SVM Classifier [31]	41	7	6	42	0.85
Wide protocol. Cohen’s d. Coarse Gaussian SVM Classifier [31]	36	12	6	42	0.75
Wide protocol. Cohen’s d. FFNN 5 neurons hidden layer [31]	46	2	5	43	0.95
Wide protocol. Cohen’s d. FFNN 10 neurons hidden layer [31]	47	1	1	47	0.98
Wide protocol. Cohen’s d. FFNN 15 neurons hidden layer [31]	47	1	2	46	0.97
Wide protocol. Cohen’s d. Convolutional Neural Network	48	0	0	48	1

## Data Availability

The data used to support the findings of this study are available from the corresponding author upon request.
